# Hip-Preserved Reconstruction Using a Customized Cementless Intercalary Endoprosthesis With an Intra-Neck Curved Stem in Patients With an Ultrashort Proximal Femur: Midterm Follow-Up Outcomes

**DOI:** 10.3389/fbioe.2022.795485

**Published:** 2022-02-28

**Authors:** Qi You, Minxun Lu, Li Min, Yuqi Zhang, Jie Wang, Yitian Wang, Chuanxi Zheng, Yong Zhou, Chongqi Tu

**Affiliations:** ^1^ Department of Orthopedics, West China Hospital, Sichuan University, Chengdu, China; ^2^ Bone and Joint 3D-Printing and Biomechanical Laboratory, Department of Orthopedics, West China Hospital, Sichuan University, Chengdu, China

**Keywords:** hip preservation, customized cementless intercalary endoprosthesis, intraneck curved stem, ultrashort proximal femur, tumor

## Abstract

**Background:** Hemiarthroplasty is widely used for proximal femoral reconstruction after tumor resection. However, complications of hemiarthroplasty include infection, hip dislocation, and acetabular wear. This study aimed to: (1) evaluate the reliability and validity of a customized cementless intercalary endoprosthesis (CCIE) with an intra-neck curved stem (INCS) to reconstruct femoral diaphyseal defects with an ultrashort proximal femur (UPF); (2) assess the lower extremity function after reconstruction with this endoprosthesis; and (3) identify the postoperative complications associated with the use of this endoprosthesis.

**Methods:** Between October 2015 and May 2019, 13 patients underwent reconstruction with a CCIE with an INCS. The distance from the center of the femoral head to the midline of the body and the apex of the acetabulum was measured preoperatively. Additionally, the distance from the tip of the INCS to the midline of the body and the apex of the acetabulum was measured postoperatively. The femoral neck–shaft angle was also measured pre- and postoperatively. After an average follow-up duration of 46 months, the radiological outcomes of the CCIE with an INCS were analyzed. Function was evaluated with the Musculoskeletal Tumor Society (MSTS) score. Pain was measured using a paper visual analog scale (VAS) pre- and postoperatively, and complications were recorded.

**Results:** Compared with our preoperative design, we found no significant difference in the postoperative distance from the tip of the INCS to the body midline (*p* = 0.187) and the apex of the acetabulum (*p* = 0.159), or in the postoperative femoral neck–shaft angle (*p* = 0.793). Thus, the INCS positions were deemed accurate. The average MSTS score was 26 (range: 24–28), and the VAS score was significantly decreased postoperatively compared with preoperatively (*p < 0.0001*). No patients developed aseptic loosening, infection, periprosthetic fracture, or prosthetic fracture as of the last follow-up.

**Conclusion:** The CCIE with an INCS was a valid and reliable method for reconstructing femoral diaphyseal defects with a UPF following malignant tumor resection. Postoperative lower extremity function was acceptable, with an appropriate individualized rehabilitation program, and the incidence of complications was low.

## Introduction

Resection of femoral diaphyseal tumors with proximal femoral extension often results in an intercalary skeletal defect with an ultrashort proximal femur (UPF) and presents a reconstructive challenge for orthopedic surgeons. If intercalary resection of a malignant bone tumor can be performed with preservation of the adjacent joints, the expected function is superior as the patient’s native joints above and below the reconstruction are left undisturbed (Fuchs et al., 2008; Panagopoulos et al., 2017; Zekry et al., 2019). Because of accurate preoperative imaging techniques, early diagnosis, and effective chemotherapy, femoral diaphyseal tumors with a long proximal femoral extension can be segmentally resected with joint preservation (Zekry et al., 2017). Alternative surgical reconstruction options include the induced membrane technique (Masquelet and Begue 2010; Accadbled et al., 2013), distraction osteogenesis (Subasi and Kapukaya 2003), autografts (Qu et al., 2015a; Houdek et al., 2017), allografts (Aponte-Tinao et al., 2018; Houdek et al., 2018), combined autografts and allografts (Capanna et al., 2007), and customized intercalary endoprostheses (Hanna et al., 2010; Stevenson et al., 2017). The induced membrane technique is an alternative method for lesions of the upper limb, and this method can prevent graft resorption and induce the secretion of growth factors. However, the induced membrane technique is a two-stage procedure and is difficult in lower limb reconstructions owing to higher stress loads (Masquelet and Begue 2010; Qu et al., 2015b). Distraction osteogenesis can achieve good biological reconstruction after successful operation; however, long external fixation time, limb bearing limitations, infection, and skin and soft tissue cutting injuries are disadvantages of this technique (Demiralp et al., 2014). Autografts with free vascularized fibular grafts (FVFG) have good bone tissue compatibility, do not undergo resorption by creeping substitution, and can incorporate into the adjacent host bone directly via bone union (Bae et al., 2005). However, FVFGs have small cross-sectional areas and are weaker than the femur. Furthermore, sufficient fibular thickness to allow full weight-bearing requires a long time to achieve hypertrophy through pressure transport, microfractures, and callus formation (Chen et al., 2007; Demiralp et al., 2014). Allografts can result in biological reconstruction of bone defects and preserve host bone stock without donor site morbidity (Bus et al., 2014). However, allografts are associated with high rates of infection, fracture, and delayed union or nonunion (Ortiz-Cruz et al., 1997). Combined autografts and allografts combine the biological activity of FVFGs with the initial mechanical strength of allografts. Moreover, FVFGs facilitate host–allograft union and minimize allograft failures (Capanna et al., 2007). However, this approach is associated with complications, such as infection, fracture, nonunion, or donor site complications. Moreover, the risk of anastomosis failure by thrombosis is a concern (Rabitsch et al., 2013).

Compared with other reconstruction methods, the available literature on femoral intercalary endoprostheses is scarce. Reconstruction with a customized femoral intercalary endoprosthesis avoids prolonged immobilization compared with autograft and allograft reconstructions and provides immediate stability, early weight-bearing, rapid rehabilitation, short hospital stay, and the ability to tolerate chemotherapy and radiotherapy after incisional healing. However, several complications related to customized endoprosthetic replacement are frequently encountered, namely aseptic loosening, mechanical failure, infection, and periprosthetic fracture (Dieckmann et al., 2014; Qu et al., 2015a). Especially in the reconstruction of femoral diaphyseal defects with a UPF, the contact area between the endoprosthetic stem and cancellous bone is insufficient. Additionally, the trochanteric region contains inadequate cancellous bone, which effects cement interdigitation for cemented femoral intercalary endoprostheses.

In our institution, we defined a UPF as the length of the residual proximal femur of ≤80 mm (the length from the pyriform fossa to the osteotomy level). For femoral diaphyseal defects with a UPF, there is currently no standard treatment choice. To make the endoprosthetic stem better match the curvature of the residual proximal femur, we use a customized cementless intercalary endoprosthesis (CCIE) with an intra-neck curved stem (INCS) to reconstruct femoral diaphyseal defects with a UPF. To our knowledge, the clinical results of using a CCIE with an INCS for reconstructing femoral diaphyseal defects with a UPF have not been evaluated previously. Therefore, the aims of this study were 1) evaluate the reliability and validity of a CCIE with an INCS to reconstruct femoral diaphyseal defects with an UPF; 2) assess the lower extremity function after reconstruction with this endoprosthesis, and 3) identify the postoperative complications associated with the use of this endoprosthesis.

## Materials and Methods

### Ethical Considerations

The retrospective study was conducted and approved by the ethics committee of the author’s institution. Each participant provided written informed consent to participate in this study.

### Patients

**FIGURE 1:**
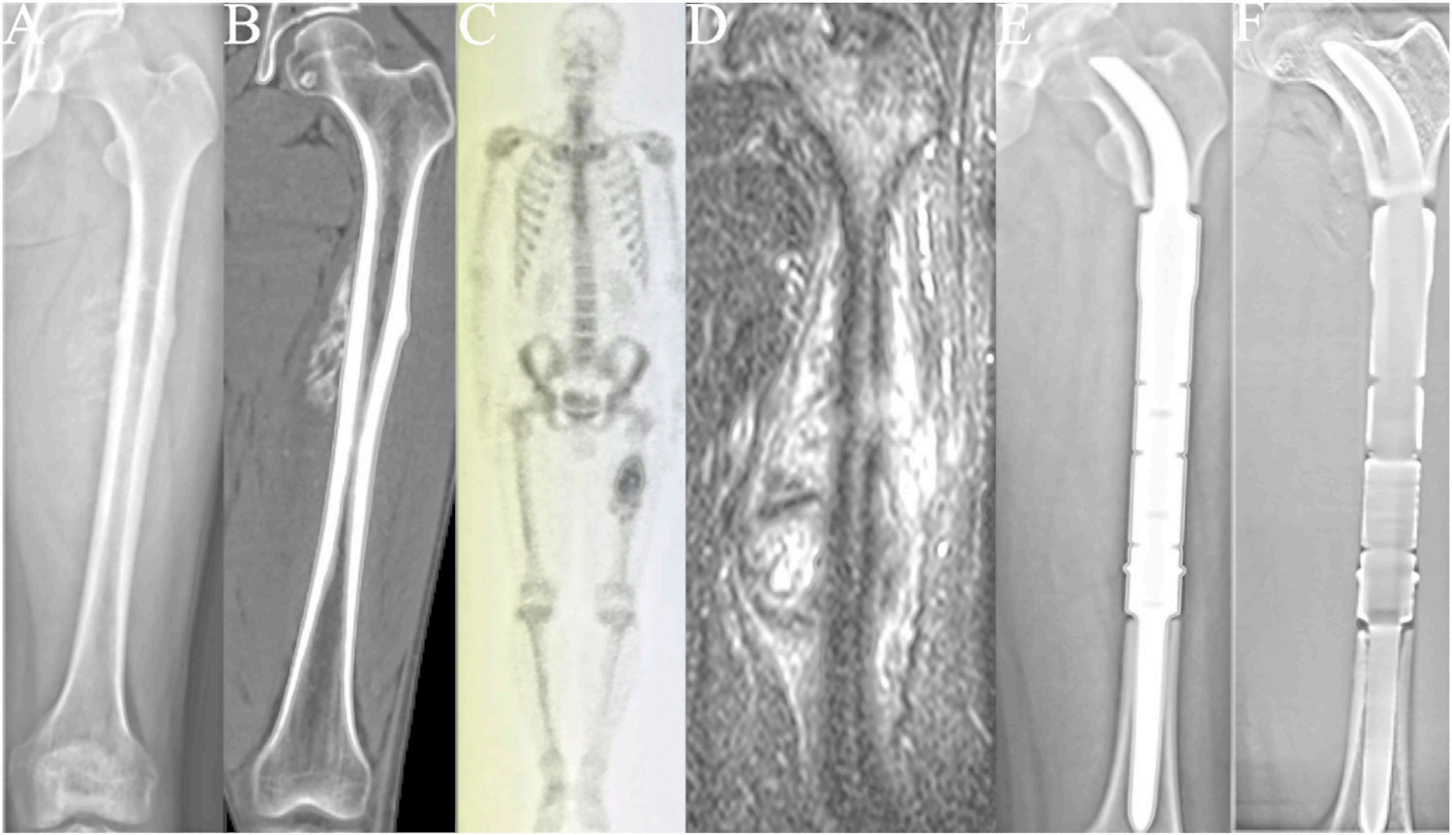
All patients underwent *en bloc* tumor resection followed by reconstruction with the customized cementless intercalary endoprosthesis with an intra-neck curved stem. **(A)** Anteroposterior radiograph of the left femur of a patient with a femoral diaphyseal osteosarcoma. **(B)** Computed tomography (CT) image of the left femur. **(C)** Single-photon emission whole-body CT image. **(D)** Magnetic resonance image of the patient’s left upper leg. **(E)** Postoperative radiograph of the femur. **(** **F)** Postoperative tomosynthesis with Shimadzu Metal Artefact Reduction Technology (T-smart) of the femur 7 days after surgery showing stable femoral diaphyseal reconstruction.

Between October 2015 and May 2019, 13 patients underwent reconstruction with a CCIE with an INCS. After an average follow-up duration of 46 months (range: 24–67 months), one patient died of lung metastases, and the remaining 12 patients were still alive. The surgical stage was determined according to the Enneking bone and soft tissue sarcoma staging system (Enneking 1986) (Table 1). Biopsy was performed for each patient before undergoing definitive surgery. The length of the required bone resection was measured by preoperative X-ray, computed tomography (CT), single-photon emission CT, and magnetic resonance imaging (Figure 1). The patients’ clinical characteristics, namely age, sex, tumor size, defect length, and the length of the residual proximal femur were collected.

**TABLE 1:** Surgical indications and stage of disease.

Patient no.	Age	Sex	Diagnosis	Metastasis	Enneking stage	Indication
1	47	M	Osteosarcoma	0	IIB	Primary sarcoma
2	34	F	Osteosarcoma	0	IIB	Primary sarcoma
3	46	F	Chondrosarcoma	0	IIB	Primary sarcoma
4	16	M	Osteosarcoma	0	IIB	Primary sarcoma
5	10	M	Osteosarcoma	Lung	IIIB	Primary sarcoma
6	14	M	Ewing sarcoma	0	IIB	Primary sarcoma
7	34	F	Osteosarcoma	0	IIB	Primary sarcoma
8	13	F	Ewing sarcoma	0	IIB	Primary sarcoma
9	24	M	Myofibroblastic sarcoma	0	IIB	Primary sarcoma
10	20	M	Ewing sarcoma	0	IIB	Primary sarcoma
11	18	F	Chondrosarcoma	0	IIB	Primary sarcoma
12	16	M	Osteosarcoma	0	IIB	Primary sarcoma
13	14	M	Ewing sarcoma	0	IIB	Primary sarcoma

M, male; F, female.

### Stem Design and Fabrication

The stem was designed to be arc-shaped. The center of the stem was a solid structure, the medial porosity of the stem was 50%, and the lateral porosity was 70% (Supplementary Figure S1). Two fins and a lock hole were designed in the curved stem. The diameter of the base of the stem was equal to the diameter of the inner surfaces of the femoral cortices. To maintain strength, the diameter of the curved stem was gradually reduced between the intertrochanteric region and femoral head-neck junction, where the diameter of the stem was about 2/3 the diameter of the medullary cavity. The curvature of the curved stem was mainly based on the medial cortex of the femoral neck and made approximately change. The diameter of the end of the curved stem was greater than 10 mm. The tip of the curved stem was designed in a “beak shape” (Supplementary Figure S2).

All INCSs were designed by our clinical team and fabricated by Chunlizhengda Medical Instruments (Tongzhou, Beijing, China). Three-dimensional CT image files were imported to Mimics V20.0 software (Materialise Corp., Leuven, Belgium) to build three-dimensional tumor and femoral models. The tumor margin was determined by CT, magnetic resonance imaging, and single-photon emission CT based on a three-dimensional model, then the osteotomy plane was obtained. According to the anatomical shape of the proximal femur, the preliminary shape of the INCS was designed using SOLIDWORKS software (Dassault Systèmes SOLIDWORKS Corp., Waltham, MA, United States). Next, the shape and appearance of the INCS was optimized using Geomagic Studio software (Geomagic Inc., Morrisville, NC, United States). Finally, the INCS was separated and generated in Magic V20 software (Materialise Corp.). The INCS models were saved as stereolithography files and imported to Mimics to simulate implantation. Endoprostheses were fabricated by electron beam melting technology (Arcam Q10plus; Arcam, Mölndal, Sweden), and the plastic trial models were fabricated with the stereolithography appearance technique (UnionTech Lite 450HD; UnionTech, Shanghai, China).

### Surgical Technique

**FIGURE 2:**
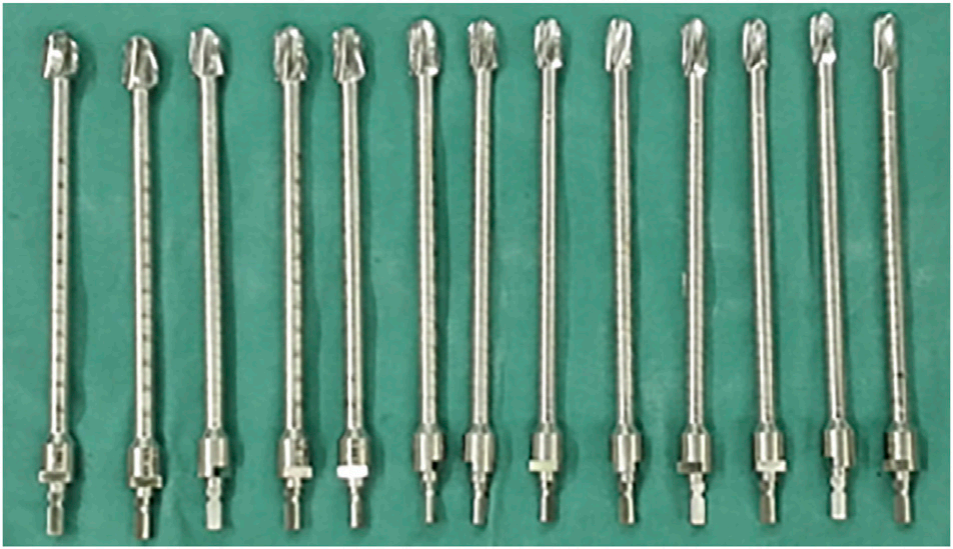
The flexible reamers of different diameters that we used in this study.

All operations were performed by the same senior surgeon (Chongqi Tu). The patients were placed in lateral recumbency, and a lateral approach was used in all cases. Tumors were resected *en bloc*, and soft tissue was removed in accordance with the results of the preoperative simulation. The degree of the osteotomy plane was controlled exactly to minimize the potential for a misfit between the customized curved stem and the residual proximal femur. After *en bloc* resection of the tumor, cancellous bone was removed from the osteotomy surface with a bone curette. The tip of the customized guide needle was pressed into the center of the femoral head with a mobile C-arm. We then used a flexible reamer with different diameters (Figure 2) with the customized guide needle in the center to gradually enlarge the medullary cavity of the residual proximal femur. To minimize bone loss and maximize primary stability of the prosthesis, the residual proximal femur was under-reamed by 0.5 mm. Thereafter, to achieve a stable press-fit, additional reaming at 0.5-mm increments was performed if needed. The direction of the femoral head was marked before the osteotomy; thus, rotation was controlled using the position of the femoral head marks as a guide when the endoprosthesis was implanted. We fit a plastic trial model smaller than the endoprosthesis first to verify that the residual proximal femur and the INCS matched. The correctly-sized endoprosthesis was then implanted, and the previously harvested cancellous bone was grafted.

### Postoperative Management

After surgery, the patients were routinely given prophylactic intravenous antibiotics for 48 h. The rehabilitation program was designed according to the surgeon’s intraoperative assessment. Generally, patients underwent bed rest for 2–3 weeks. The lower extremity was maintained in a neutral position, and knee and ankle flexion and extension exercises were performed in bed during this period. Partial weight-bearing was initiated using two crutches after week 3, and hip flexion and abduction exercises were initiated after week 4. Partial walking weight-bearing, using one crutch, was allowed after week 8. Progression to full weight-bearing was initiated after week 12.

As per our protocol, follow-up assessments were performed monthly during the first 3 months, then every 3 months for 2 years, and then yearly. Patients received a physical examination of their affected extremity at each follow-up visit. Pain was measured using a paper visual analog score (VAS). Radiographic assessment was performed monthly during the first 3 months, then every 3 months for the first year, every 6 months for the second year, and then annually. The Musculoskeletal Tumor Society (MSTS) scoring system was used to assess lower limb function (Enneking et al., 1993). The distance from the tip of the INCS to the body midline and the top of the acetabulum was measured postoperatively with X-rays. The femoral neck–shaft angle was also measured pre- and postoperatively. The condition of the bone–prosthesis interface was evaluated by X-ray, CT, and T-smart. Bone ingrowth into the prosthesis was evaluated by radiographic variables, namely, bone bridging, spot welding, and neocortex formation. Complications related to INCS implantation were assessed, namely aseptic loosening, prosthetic fracture, infection, and periprosthetic fracture.

### Statistical Analysis

We used the paired *t* test to assess the differences between the pre- and postoperative measurements. A *p*-value of ≤0.05 was considered statistically significant, and SPSS software (version 19.0; IBM Corp., Armonk, NY, United States) was used for the data analysis.

## Results

### Radiographic Analysis

**FIGURE 3:**
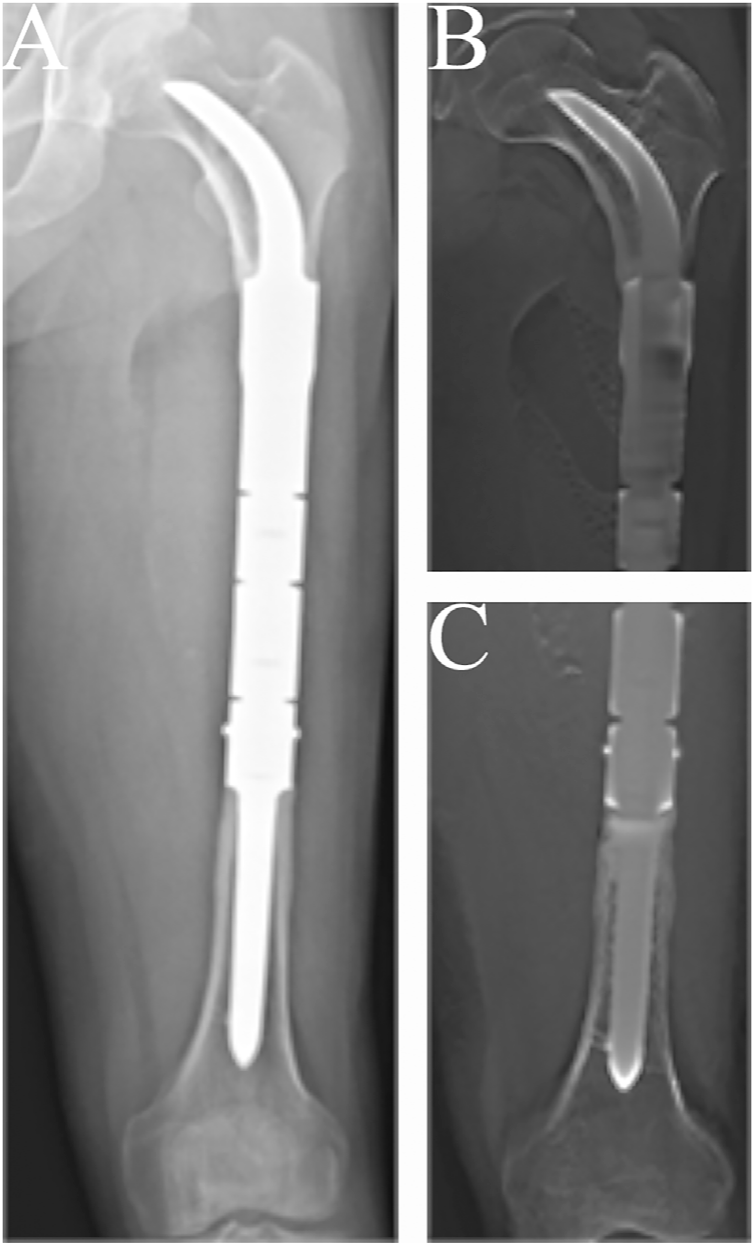
Radiographs showing the 57-months postoperative views of the customized cementless intercalary endoprosthesis with an intra-neck curved stem placed during treatment for an osteosarcoma. **(A)** Posteroanterior radiograph of the entire femur. **(B)** Posteroanterior tomosynthesis with Shimadzu Metal Artefact Reduction Technology (T-smart) views of the stem insertion region in the proximal femur. **(C)** Posteroanterior T-smart views of the stem insertion region in the distal femur.

**FIGURE 4:**
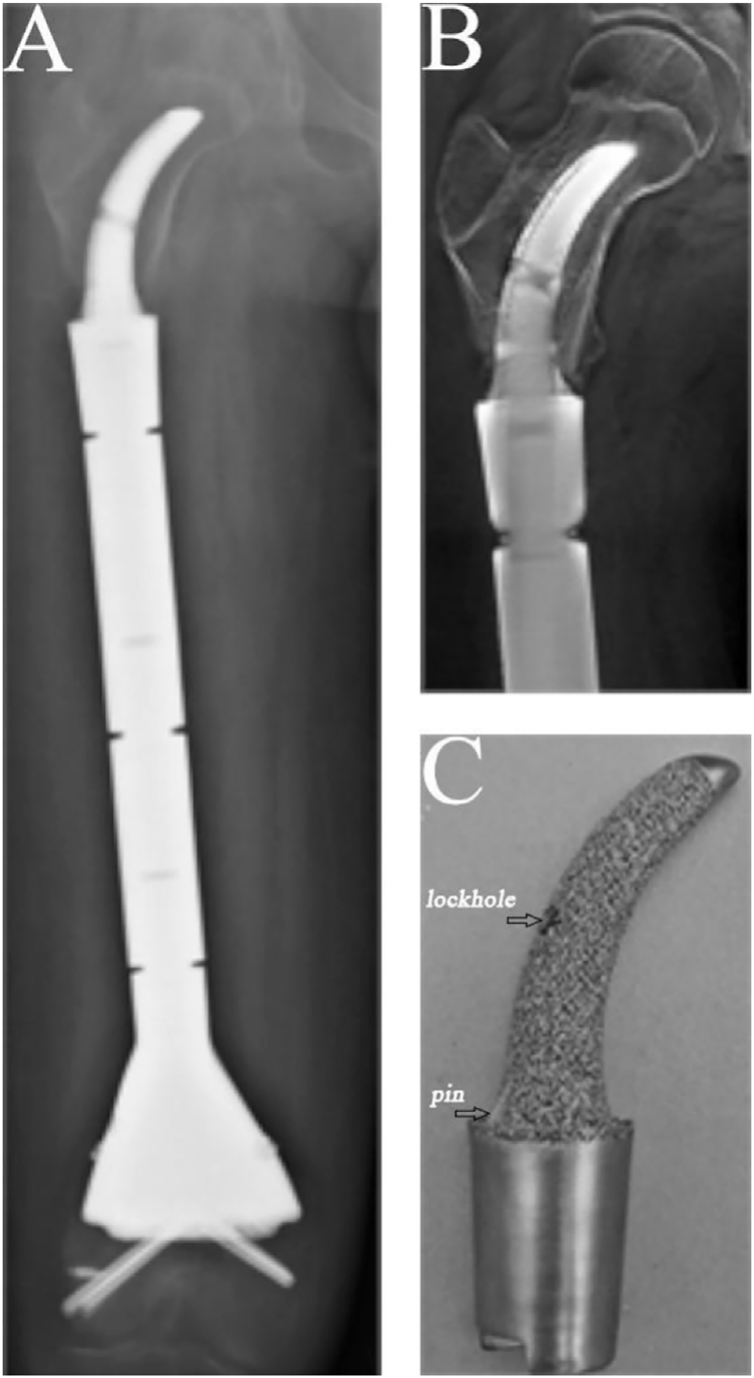
A case of reconstruction of the femoral diaphysis following femoral diaphyseal resection of 73% of the length of the femur. **(A)** Posteroanterior radiograph of the entire femur. **(B)** Posteroanterior T-smart view of the stem insertion region of the femur. **(C)** Gross appearance of the curved stem.

The condition of the bone–prosthesis interface was evaluated by X-ray and T-smart. No fretting wear around the endoprosthetic stem was found in the enrolled patients. We also found no obvious radiolucent line, and radiographic signs of bone ingrowth on the bone–stem interface were found in all stems. A typical case of postoperative neocortex formation is shown in Figure 3. In one patient, the CCIE with an INCS was used in the femoral diaphyseal defect reconstruction after resecting >70% of the femoral length owing to massive tumor resection, and a stable bone–prothesis interface was achieved at the last follow-up (Figure 4). Compared with the distance from the center of the femoral head to the midline of the body and the apex of the acetabulum preoperatively, the distance from the tip of the INCS to the midline of the body (*p* = 0.187) and the apex of the acetabulum (*p* = 0.159) was not statistically significant postoperatively. Furthermore, the femoral neck–shaft angle did not differ significantly between pre- and postoperative values (*p* = 0.793) (Table 2). Thus, the INCS positions were deemed accurate.

**TABLE 2:** Details of the surgical technique and INCS location evaluation.

Patient no	Length of femur resection, mm	Percentage of femur resection length in the total femur length, %	Length of residual proximal femur, mm	d_1_, mm (preoperative/postoperative)	d_2_, mm (preoperative/postoperative)	Neck-shaft angle, ° (preoperative/postoperative
1	180.00	43.16	71.90	90.30/93.50	27.50/30.50	124/120
2	79.80	19.10	54.20	89.10/92.00	22.80/23.20	128/123
3	98.30	21.57	79.20	91.50/93.20	23.50/24.50	122/127
4	285.00	73.08	53.60	66.80/68.10	28.00/30.50	130/125
5	211.30	51.66	53.60	81.60/79.50	21.80/22.50	129/126
6	248.00	58.35	72.50	89.50/91.20	24.20/25.80	122/125
7	86.70	21.58	77.30	89.60/91.70	25.50/26.90	127/122
8	128.60	30.85	76.40	91.40/92.30	23.60/18.70	128/134
9	116.70	26.20	74.70	92.80/91.00	25.80/21.30	122/123
10	185.00	39.78	78.00	91.20/92.10	26.20/29.80	125/119
11	136.80	28.93	75.60	94.00/94.50	28.20/30.90	119/124
12	159.70	39.75	69.50	91.30/89.60	25.80/28.70	130/125
13	137.60	32.11	69.60	94.20/93.50	28.90/34.80	120/128

d_1_, Preoperative distance from the center of the femoral head to the midline of the body/postoperative distance from the tip of the INCS to the midline of the body; d_2_, Preoperative distance from the center of the femoral head to the apex of the acetabulum/postoperative distance from the tip of the INCS to the apex of the acetabulum; INCS, intra-neck curved stem.

### Function

Regarding lower extremity function, the average MSTS score was 26 (range: 24–28) at the last follow-up. None of the surviving patients with femoral diaphyseal reconstruction required crutches or other walking aids at the last follow-up. One patient complained of pain in the lower extremity when walking unsupported for distances longer than 5,000 m; this patient had a VAS score of two at final follow-up, but had no imaging complications associated with the use of this endoprosthesis. Compared with the preoperative VAS score, the postoperative score decreased significantly (*p < 0.0001*) (Table 3). No pain or Trendelenburg gait was found in the other patients at the last follow-up.

**TABLE 3:** Results for patients undergoing femoral reconstruction with an intra-neck curved stem endoprosthesis.

Patient no	Oncological status	Follow-up (months)	Complication	VAS (preoperative/postoperative)	MSTS
1	NED	57	None	6/0	27
2	NED	30	None	6/0	24
3	NED	67	None	5/0	27
4	NED	24	None	7/0	25
5	DOD	27	None	—	—
6	NED	55	None	6/0	26
7	NED	63	None	5/0	28
8	NED	48	None	6/2	25
9	NED	51	None	7/0	25
10	NED	36	None	5/0	26
11	NED	55	None	4/0	27
12	NED	44	None	6/0	26
13	NED	41	None	5/0	26

VAS, visual analog scale; NED, no evidence of disease; DOD, died of disease; MSTS, musculoskeletal tumor society.

### Complications

No patients developed periprosthetic infection, implant fracture, periprosthetic fracture, nerve palsy, or vascular incidents. All of the endoprostheses were well-osseointegrated, no aseptic loosening was observed in this series.

## Discussion

The optimal treatment method for femoral diaphyseal tumors with proximal metaphyseal extension is controversial. Total femoral replacement is an alternative surgical option; however, this approach is associated with dislocation, structural failures, and soft tissue failures (Sevelda et al., 2015). In addition, proximal femoral resection often results in an additional compartment being opened. Once local recurrence or infection occurs, the hip joint may be contaminated. For contaminated hip joints, eventual hemipelvectomy may be necessary to achieve extensive margins to prevent local recurrence (Kalra et al., 2010). Therefore, preserving the hip joint is important for improving functional lower limb outcomes, and preserving bone stock is necessary for possible future revision.

Benevenia et al. (2016) reported the mid-term results of cemented endoprosthetic replacement in femoral intercalary resection; 12 of 21 patients (57%) with femoral reconstructions developed complications of spacer failure, infection, or aseptic loosening. For cemented straight femoral endoprostheses, it is difficult to provide lasting fixation between the bone and the endoprosthesis because of the insufficient residual proximal femoral length. In addition, with a UPF, the proximal endpoint of the straight intramedullary stem is often located in the trochanteric region; however, the trochanteric region not only has a large offset but also contains inadequate cancellous bone (Unwin et al., 1996). Lack of cancellous bone would affect the interdigitation of bone cement (Ebramzadeh et al., 1994). Currently, the promising cementless short-stem solutions are the Compress^®^ implant (Calvert et al., 2014) and the Buxtehude stem (Dieckmann et al., 2014). The advantage of the Compress^®^ implant is that the associated compressive osteointegration avoids stress shielding and saves bone stock; however, the early aseptic loosening rate ranges from 3.8 to 14% (Pedtke et al., 2012; Calvert et al., 2014). The Buxtehude stem was used in a study of patients with a UPF, in which the early aseptic loosening rate was 12.5%, and fixation screw breakage occurred during follow-up (Dieckmann et al., 2014).

In our study, no obvious radiolucent lines between the implant and the bone were found on the proximal femur on mediolateral or anteroposterior X-ray views. Moreover, bone ingrowth on the bone–stem interface was found for all stems. In addition, compared with previous studies (Calvert et al., 2014; Dieckmann et al., 2014) of reconstructing femoral diaphyseal defects with a UPF, the rate of aseptic loosening in our study was low. We believe the reasons are as follows: (1) The endoprosthesis we used to reconstruct femoral diaphyseal defects with a UPF was a good fit with the anatomy of the proximal femur, and the INCS positions were deemed accurate postoperatively. (2) The INCS reduces the offset distance between the long axis of the femur and a line passing through the center of the femoral head and condyles, which is considered a measure of the bending moment about a dorsoventral axis at any level (Unwin et al., 1996). Compared with a straight stem, the offset distance of an INCS is smaller; therefore, the bending moment is also smaller, which may be an important factor in decreasing the rate of aseptic loosening of the endoprosthesis (Wyatt et al., 2019). (3) The INCS is coated with hydroxyapatite or 3D-printed porous titanium, which can facilitate biological bone ingrowth at the bone–prosthesis interface (Van der Stok et al., 2013; Lu et al., 2018; Zhao et al., 2020). (4) The curved stem we used has two fins, symmetrically arranged in the medial and lateral planes at the base of the stem, providing guidance for implantation and an additional derotational force (Figure 4C).

Bone resorption at the lateral junction part of implant-bone be found in two patients at the last follow-up. In our opinion, the lateral junction part of the implant-bone was the side of tensile stress. Good osseointegration was achieved between the curved stem and host bone and the tensile stress of the lateral junction part of the implant-bone was possibly reduced. Thus, stress shielding occurred at the lateral junction part, so the bone resorption appeared there. At present, bone resorption is less in the lateral junction part and the remaining cortical bone can still meet the tensile stress requirement. Furthermore, the medial cortical bone can provide partial support when the stability of the curved stem is good. It remains to be explored whether bone resorption will increase in the future.

The average postoperative lower extremity function (MSTS) score in the surviving patients in this study was 26 points, which is superior to that reported in other studies (Calvert et al., 2014; Dieckmann et al., 2014; Zheng et al., 2019). Although rehabilitation of lower extremity function was incomplete, patients experienced adequate pain relief and self-care limb function. In particular, the patients’ focus often shifted from considering themselves “sarcoma survivors” to “functionally recovered” after surgery. The short postoperative recovery time and early full weight-bearing are particularly valuable. In addition, our patients experienced no limitations on lower limb function in daily life, postoperatively. The reasons are as follows: 1) preserving the hip joint helps reduce surgical disruption and minimizes muscle damage; therefore, maximal restoration of lower extremity function is possible; 2) endoprosthesis stability, natural bodyweight transmission, and good bone–endoprosthesis interface integration benefitted the restoration of limb function; and 3) the rehabilitation program allowed early functional training, resulting in better lower extremity function.

Periprosthetic infection and periprosthetic fracture are common complications after reconstruction of the distal femur or diaphysis (Grimer et al., 2002; Gosheger et al., 2006; Choi et al., 2016; Bus et al., 2017). At the last follow-up, in our study, we observed no periprosthetic infection or periprosthetic fracture. In our opinion, periprosthetic infection for oncologic patients may be related to soft tissue coverage, immune-compromising treatments, duration of the procedure, and extensive surgical dissection. No infections occurred in our study, and we believe the reasons are as follows: First, soft tissue was lavaged with abundant normal saline intraoperatively. Second, we paid attention to each patient’s condition, the use of antibiotics, and postoperative drainage. No patients developed periprosthetic fracture intraoperatively. To avoid periprosthetic fracture, all operations were performed by the same senior surgeon, and the osteotomy plane was precisely controlled to minimize the risk of a misfit between the curved stem and the residual proximal femur. Additionally, when enlarging the medullary cavity of the residual proximal femur, the residual proximal femur was under-reamed by 0.5 mm followed by additional 0.5-mm reaming, as needed, to achieve a stable press-fit. No aseptic loosening was found in this series at the last follow-up. To reduce aseptic loosening, the biocompatibility of the endoprosthesis was enhanced by improving the matching degree at the contact area and modifying the configuration of the endoprosthesis (Palmquist et al., 2013; Hara et al., 2016; Ghouse et al., 2019).

We acknowledge some limitations in this study. First, our follow-up time was insufficient to verify the long-term efficacy of the curved stem. Unknown shortcomings might occur in long-term follow-up. Second, the retrospective, non-comparative design and small sample size limited the power of this study because prosthetic reconstructions for femoral diaphyseal defects following malignant tumor resection are rare.

## Conclusion

This study presented the preliminary results of using a CCIE with an INCS, which showed proof of principle for the application of an INCS. In conclusion, an INCS represents a feasible treatment option for femoral diaphyseal defects with a UPF. The main advantages of this treatment are early weight-bearing, low complication rate, and good lower limb functional outcomes.

## Data Availability

The original contributions presented in the study are included in the article/Supplementary Material, further inquiries can be directed to the corresponding authors.

## Supplementary Material

Supplementary Figure S1 Schematic diagram of the curved stem. **(A)** The positional relationship of the curved stem and bone. **(B)** The porosity of the curved stem.

Supplementary Figure S2 Schematic diagram of different cross section of the curved stem. **(A)** The base of the curved stem. **(B)** The middle part of the curved stem. **(C)** The end of the curved stem. **(D)** The tip of the curved stem.

## References

[B2] Accadbled F. Mazeau P. Chotel F. Cottalorda J. Sales de Gauzy J. Kohler R. (2013). Induced-Membrane Femur Reconstruction after Resection of Bone Malignancies: Three Cases of Massive Graft Resorption in Children. Orthopaedics Traumatol. Surg. Res. 99 (4), 479–483. 10.1016/j.otsr.2013.01.008 23608487

[B3] Aponte-Tinao L. A. Albergo J. I. Ayerza M. A. Muscolo D. L. Ing F. M. Farfalli G. L. (2018). What Are the Complications of Allograft Reconstructions for Sarcoma Resection in Children Younger Than 10 Years at Long-Term Followup? Clin. Orthop. Relat. Res. 476 (3), 548–555. 10.1007/s11999.0000000000000055 29529639 PMC6260017

[B4] Bae D. S. Waters P. M. Sampson C. E. (2005). Free Use of Free Vascularized Fibular Graft for Congenital Ulnar Pseudarthrosis Surgical Decision Making in the Growing Child. J. Pediatr. Orthop. 25 (6), 755–762. 10.1097/01.bpo.0000186241.29415.df 16294132

[B5] Benevenia J. Kirchner R. Patterson F. Beebe K. Wirtz D. C. Rivero S. (2016). Outcomes of a Modular Intercalary Endoprosthesis as Treatment for Segmental Defects of the Femur, Tibia, and Humerus. Clin. orthopaedics Relat. Res. 474 (2), 539–548. 10.1007/s11999-015-4588-z PMC4709281 26475032

[B6] Bus M. P. A. Dijkstra P. D. S. van de Sande M. A. J. Taminiau A. H. M. Schreuder H. W. B. Jutte P. C. (2014). Intercalary Allograft Reconstructions Following Resection of Primary Bone Tumors. The J. bone Jt. surgeryAmerican volume 96 (4), e26. 10.2106/JBJS.M.00655 24553895

[B7] Bus M. P. A. van de Sande M. A. J. Fiocco M. Schaap G. R. Bramer J. A. M. Dijkstra S. P. D. (2017). What Are the Long-Term Results of MUTARS Modular Endoprostheses for Reconstruction of Tumor Resection of the Distal Femur and Proximal Tibia? Clin. orthopaedics Relat. Res. 475 (3), 708–718. 10.1007/s11999-015-4644-8 PMC5289150 26649558

[B8] Calvert G. T. Cummings J. E. Bowles A. J. Jones K. B. Wurtz D. L. Randall L. R. (2014). A Dual-Center Review of Compressive Osseointegration for Fixation of Massive Endoprosthetics: 2- to 9-year Followup. Clin. orthopaedics Relat. Res. 472 (3), 822–829. 10.1007/s11999-013-2885-y PMC3916600 23467985

[B10] Capanna R. Campanacci D. A. Belot N. Beltrami G. Manfrini M. Innocenti M. (2007). A New Reconstructive Technique for Intercalary Defects of Long Bones: the Association of Massive Allograft with Vascularized Fibular Autograft. Long-Term Results and Comparison with Alternative Techniques. Orthop. Clin. North America 38 (1), 51–60. 10.1016/j.ocl.2006.10.008 17145294

[B11] Chen C. M. Disa J. J. Lee H.-Y. Mehrara B. J. Hu Q.-Y. Nathan S. (2007). Reconstruction of Extremity Long Bone Defects after Sarcoma Resection with Vascularized Fibula Flaps: A 10-year Review. Plast. Reconstr. Surg. 119 (3), 915–924. 10.1097/01.prs.0000252306.72483.9b 17312496

[B12] Choi H.-S. Nho J.-H. Kim C.-H. Kwon S.-W. Park J.-S. Suh Y.-S. (2016). Revision Arthroplasty Using a MUTARS Prosthesis in Comminuted Periprosthetic Fracture of the Distal Femur. Yonsei Med. J. 57 (6), 1517–1522. 10.3349/ymj.2016.57.6.1517 27593884 PMC5011288

[B13] Demiralp B. Ege T. Kose O. Yurttas Y. Basbozkurt M. (2014). Reconstruction of Intercalary Bone Defects Following Bone Tumor Resection with Segmental Bone Transport Using an Ilizarov Circular External Fixator. J. Orthopaedic Sci. 19 (6), 1004–1011. 10.1007/s00776-014-0632-1 25146001

[B14] Dieckmann R. Henrichs M.-P. Gosheger G. Höll S. Hardes J. Streitbürger A. (2014). Short-Stem Reconstruction for Megaendoprostheses in Case of an Ultrashort Proximal Femur. BMC Musculoskelet. Disord. 15, 190. 10.1186/1471-2474-15-190 24885859 PMC4067112

[B16] Ebramzadeh E. Sarmiento A. McKellop H. A. Llinas A. Gogan W. (1994). The Cement Mantle in Total Hip Arthroplasty. Analysis of Long-Term Radiographic Results. The J. Bone Jt. SurgeryAmerican volume 76 (1), 77–87. 10.2106/00004623-199401000-00010 8288668

[B17] Enneking W. F. Dunham W. Gebhardt M. C. Malawar M. Pritchard D. J. (1993). A System for the Functional Evaluation of Reconstructive Procedures after Surgical Treatment of Tumors of the Musculoskeletal System. Clin. orthopaedics Relat. Res. 286, 241–246. 10.1097/00003086-199301000-00035 8425352

[B18] Enneking W. F. (1986). A System of Staging Musculoskeletal Neoplasms. Clin. Orthop. Relat. Res. 204, 9–24. 10.1097/00003086-198603000-00003 3456859

[B19] Fuchs B. Ossendorf C. Leerapun T. Sim F. H. (2008). Intercalary Segmental Reconstruction after Bone Tumor Resection. Eur. J. Surg. Oncol. (Ejso) 34 (12), 1271–1276. 10.1016/j.ejso.2007.11.010 18191363

[B20] Ghouse S. Reznikov N. Boughton O. R. Babu S. Ng K. C. G. Blunn G. (2019). The Design and *In Vivo* Testing of a Locally Stiffness-Matched Porous Scaffold. Appl. Mater. Today 15, 377–388. 10.1016/j.apmt.2019.02.017 31281871 PMC6609455

[B21] Gosheger G. Gebert C. Ahrens H. Streitbuerger A. Winkelmann W. Hardes J. (2006). Endoprosthetic Reconstruction in 250 Patients with Sarcoma. Clin. orthopaedics Relat. Res. 450, 164–171. 10.1097/01.blo.0000223978.36831.39 16691142

[B22] Grimer R. J. Belthur M. Chandrasekar C. Carter S. R. Tillman R. M. (2002). Two-stage Revision for Infected Endoprostheses Used in Tumor Surgery. Clin. orthopaedics Relat. Res. 395, 193–203. 10.1097/00003086-200202000-00022 11937881

[B23] Hanna S. A. Sewell M. D. Aston W. J. S. Pollock R. C. Skinner J. A. Cannon S. R. (2010). Femoral Diaphyseal Endoprosthetic Reconstruction after Segmental Resection of Primary Bone Tumours. The J. Bone Jt. Surg. Br. volumeBritish volume 92-B (6), 867–874. 10.1302/0301-620X.92B6.23449 20513887

[B24] Hara D. Nakashima Y. Sato T. Hirata M. Kanazawa M. Kohno Y. (2016). Bone Bonding Strength of diamond-structured Porous Titanium-alloy Implants Manufactured Using the Electron Beam-Melting Technique. Mater. Sci. Eng. C 59, 1047–1052. 10.1016/j.msec.2015.11.025 26652463

[B25] Houdek M. T. Wagner E. R. Bishop A. T. Shin A. Y. Rose P. S. Sim F. H. (2017). Complications and Long-Term Outcomes of Free Fibula Reconstruction Following Resection of a Malignant Tumor in the Extremities. Plast. Reconstr. Surg. 139 (2), 510e–519e. 10.1097/PRS.0000000000003004 28121893

[B27] Houdek M. T. Rose P. S. Milbrandt T. A. Stans A. A. Moran S. L. Sim F. H. (2018). Comparison of Pediatric Intercalary Allograft Reconstructions with and without a Free Vascularized Fibula. Plast. Reconstr. Surg. 142 (4), 1065–1071. 10.1097/PRS.0000000000004794 30020231

[B28] Kalra S. Abudu A. Murata H. Grimer R. J. Tillman R. M. Carter S. R. (2010). Total Femur Replacement: Primary Procedure for Treatment of Malignant Tumours of the Femur. Eur. J. Surg. Oncol. (Ejso) 36 (4), 378–383. 10.1016/j.ejso.2009.11.002 20230929

[B29] Lu M. Wang J. Xiao C. Tang F. Min L. Zhou Y. (2018). Uncemented, Curved, Short Endoprosthesis Stem for Distal Femoral Reconstruction: Early Follow-Up Outcomes. World J. Surg. Onc 16 (1), 183. 10.1186/s12957-018-1486-3 PMC6131732 30200979

[B30] Masquelet A. C. Begue T. (2010). The Concept of Induced Membrane for Reconstruction of Long Bone Defects. Orthop. Clin. North America 41 (1), 27–37. 10.1016/j.ocl.2009.07.011 19931050

[B31] Ortiz-Cruz E. Gebhardt M. C. Jennings L. C. Springfield D. S. Mankin H. J. (1997). The Results of Transplantation of Intercalary Allografts after Resection of Tumors. A Long-Term Follow-Up Study. J. Bone Joint Surg. Am 79 (1), 97–106. 10.2106/00004623-199701000-00010 9010190

[B32] Palmquist A. Snis A. Emanuelsson L. Browne M. Thomsen P. (2013). Long-Term Biocompatibility and Osseointegration of Electron Beam Melted, Free-Form-Fabricated Solid and Porous Titanium alloy: Experimental Studies in Sheep. J. Biomater. Appl. 27 (8), 1003–1016. 10.1177/0731684411431857 22207608

[B33] Panagopoulos G. N. Mavrogenis A. F. Mauffrey C. Lesenský J. Angelini A. Megaloikonomos P. D. (2017). Intercalary Reconstructions after Bone Tumor Resections: A Review of Treatments. Eur. J. Orthop. Surg. Traumatol. 27 (6), 737–746. 10.1007/s00590-017-1985-x 28585185

[B34] Pedtke A. C. Wustrack R. L. Fang A. S. Grimer R. J. O’Donnell R. J. (2012). Aseptic Failure: How Does the Compress(®) Implant Compare to Cemented Stems? Clin. orthopaedics Relat. Res. 470 (3), 735–742. 10.1007/s11999-011-2159-5 PMC3270164 22045069

[B35] Qu H. Guo W. Yang R. Li D. Tang S. Yang Y. (2015b). Reconstruction of Segmental Bone Defect of Long Bones after Tumor Resection by Devitalized Tumor-Bearing Bone. World J. Surg. Onc 13, 282. 10.1186/s12957-015-0694-3 PMC4581416 26399398

[B36] Qu H. Guo W. Yang R. Tang X. Yan T. Li D. (2015a). Cortical Strut Bone Grafting and Long-Stem Endoprosthetic Reconstruction Following Massive Bone Tumour Resection in the Lower Limb. Bone Jt. J. 97-B (4), 544–549. 10.1302/0301-620X.97B4.34695 25820896

[B37] Rabitsch K. Maurer-Ertl W. Pirker-Frühauf U. Wibmer C. Leithner A. (2013). Intercalary Reconstructions with Vascularised Fibula and Allograft after Tumour Resection in the Lower Limb. Sarcoma 2013, 160295. 10.1155/2013/160295 23766665 PMC3676952

[B38] Sevelda F. Schuh R. Hofstaetter J. G. Schinhan M. Windhager R. Funovics P. T. (2015). Total Femur Replacement after Tumor Resection: Limb Salvage Usually Achieved but Complications and Failures Are Common. Clin. orthopaedics Relat. Res. 473 (6), 2079–2087. 10.1007/s11999-015-4282-1 PMC4419011 25832007

[B39] Stevenson J. D. Wigley C. Burton H. Ghezelayagh S. Morris G. Evans S. (2017). Minimising Aseptic Loosening in Extreme Bone Resections: Custom-Made Tumour Endoprostheses with Short Medullary Stems and Extra-Cortical Plates. Bone Jt. J. 99-B (12), 1689–1695. 10.1302/0301-620X.99B12.BJJ-2017-0213.R1 29212694

[B41] Subasi M. Kapukaya A. (2003). Distraction Osteogenesis for Treatment of Bone Loss in the Lower Extremity. J. Orthopaedic Sci. 8 (6), 882–884. 10.1007/s00776-003-0728-5 14648283

[B42] Unwin P. S. Cannon S. R. Grimer R. J. Kemp H. B. S. Sneath R. S. Walker P. S. (1996). Aseptic Loosening in Cemented Custom-Made Prosthetic Replacements for Bone Tumours of the Lower Limb. J. Bone Jt. Surg. Br. volumeBritish volume 78-B (1), 5–13. 10.1302/0301-620x.78b1.0780005 8898118

[B43] Van der Stok J. Van der Jagt O. P. Amin Yavari S. De Haas M. F. P. Waarsing J. H. Jahr H. (2013). Selective Laser Melting-Produced Porous Titanium Scaffolds Regenerate Bone in Critical Size Cortical Bone Defects. J. Orthop. Res. 31 (5), 792–799. 10.1002/jor.22293 23255164

[B44] Wyatt M. C. Kieser D. C. Kemp M. A. McHugh G. Frampton C. M. A. Hooper G. J. (2019). Does the Femoral Offset Affect Replacements? the Results from a National Joint Registry. HIP Int. 29 (3), 289–298. 10.1177/1120700018780318 29873253

[B45] Zekry K. M. Yamamoto N. Hayashi K. Takeuchi A. Higuchi T. Abe K. (2017). Intercalary Frozen Autograft for Reconstruction of Malignant Bone and Soft Tissue Tumours. Int. Orthopaedics (Sicot) 41 (7), 1481–1487. 10.1007/s00264-017-3446-x 28343291

[B46] Zekry K. M. Yamamoto N. Hayashi K. Takeuchi A. Alkhooly A. Z. A. Abd-Elfattah A. S. (2019). Reconstruction of Intercalary Bone Defect after Resection of Malignant Bone Tumor. J. Orthop. Surg. (Hong Kong) 27 (1), 2309499019832970. 10.1177/2309499019832970 30879390

[B47] Zhao D. Tang F. Min L. Lu M. Wang J. Zhang Y. (2020). Intercalary Reconstruction of the "Ultra-Critical Sized Bone Defect" by 3D-Printed Porous Prosthesis after Resection of Tibial Malignant Tumor. Cancer Manag. Res. 12, 2503–2512. 10.2147/CMAR.S245949 32308487 PMC7152541

[B48] Zheng K. Yu X.-C. Hu Y.-C. Shao Z.-W. Xu M. Wang B.-C. (2019). Outcome of Segmental Prosthesis Reconstruction for Diaphyseal Bone Tumors: A Multi-Center Retrospective Study. BMC cancer 19 (1), 638. 10.1186/s12885-019-5865-0 31253134 PMC6599373

