# Supplementary figures and images for: Hip-Preserved Reconstruction Using a Customized Cementless Intercalary Endoprosthesis With an Intra-Neck Curved Stem in Patients With an Ultrashort Proximal Femur: Midterm Follow-Up Outcomes
Source: Front Bioeng Biotechnol. 2022 Feb 28;10:795485. doi: 10.3389/fbioe.2022.795485 (PMC8918842; doi:10.3389/fbioe.2022.795485)

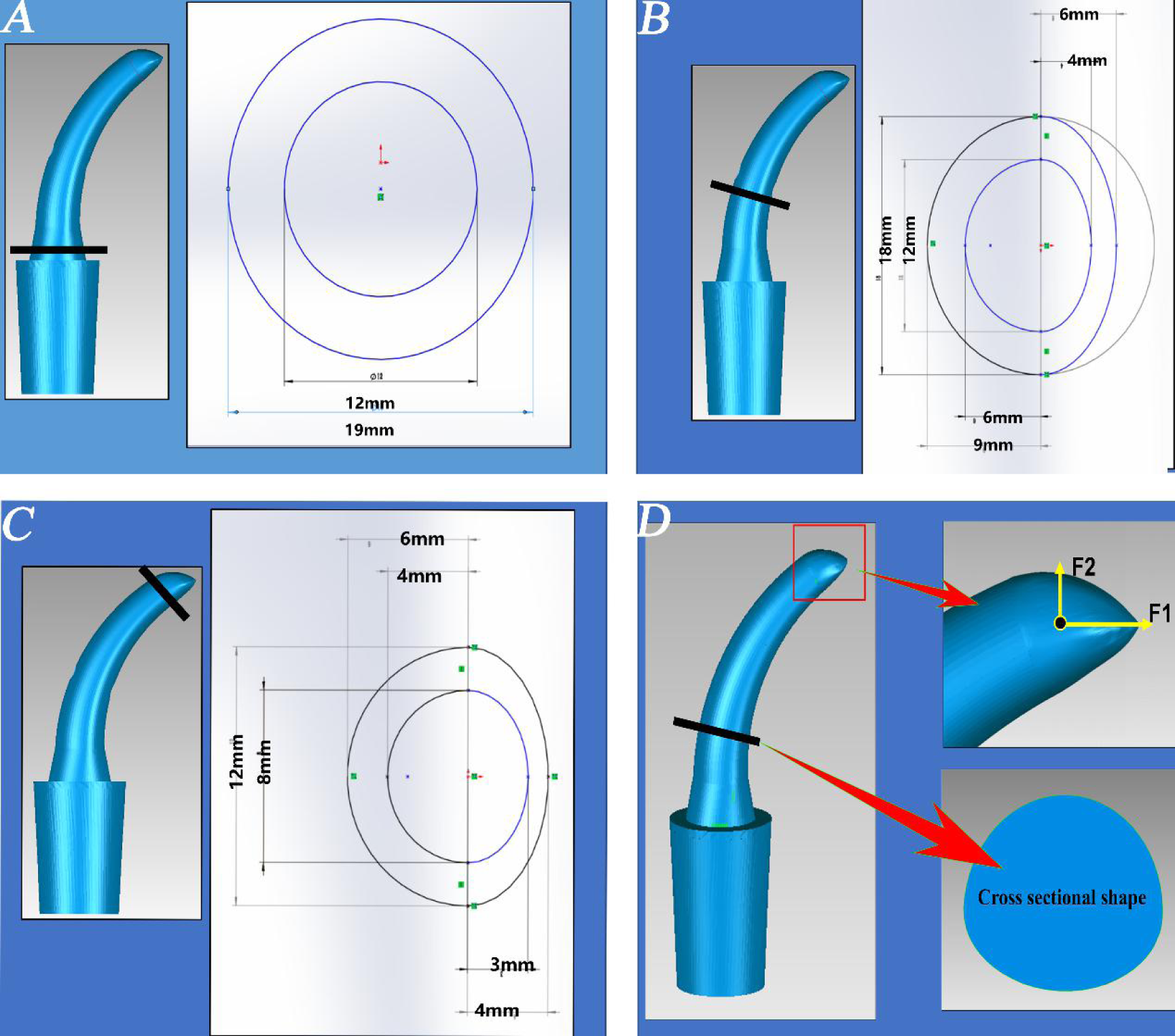

Supplement: Supplementary file 1 [file Image2.TIF]

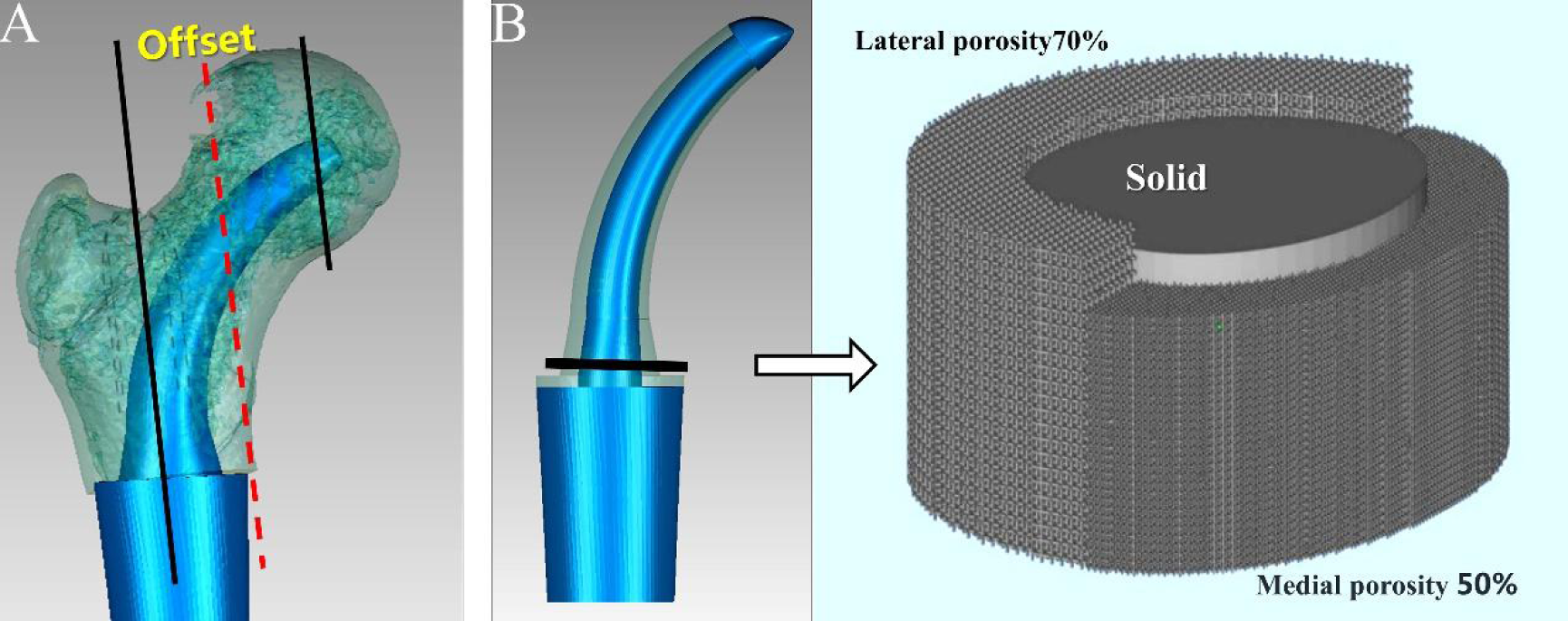

Supplement: Supplementary file 2 [file Image1.TIF]
